# Drug Repositioning for P-Glycoprotein Mediated Co-Expression Networks in Colorectal Cancer

**DOI:** 10.3389/fonc.2020.01273

**Published:** 2020-08-13

**Authors:** Hande Beklen, Gizem Gulfidan, Kazim Yalcin Arga, Adil Mardinoglu, Beste Turanli

**Affiliations:** ^1^Department of Bioengineering, Marmara University, Istanbul, Turkey; ^2^Centre for Host–Microbiome Interactions, Faculty of Dentistry, Oral & Craniofacial Sciences, King's College London, London, United Kingdom; ^3^Science for Life Laboratory, KTH—Royal Institute of Technology, Stockholm, Sweden; ^4^Department of Bioengineering, Istanbul Medeniyet University, Istanbul, Turkey

**Keywords:** colorectal cancer, drug repositioning, multi-drug resistance, P-glycoprotein, co-expression networks, multi-drug resistance protein

## Abstract

Colorectal cancer (CRC) is one of the most fatal types of cancers that is seen in both men and women. CRC is the third most common type of cancer worldwide. Over the years, several drugs are developed for the treatment of CRC; however, patients with advanced CRC can be resistant to some drugs. P-glycoprotein (P-gp) (also known as Multidrug Resistance 1, MDR1) is a well-identified membrane transporter protein expressed by ABCB1 gene. The high expression of MDR1 protein found in several cancer types causes chemotherapy failure owing to efflux drug molecules out of the cancer cell, decreases the drug concentration, and causes drug resistance. As same as other cancers, drug-resistant CRC is one of the major obstacles for effective therapy and novel therapeutic strategies are urgently needed. Network-based approaches can be used to determine specific biomarkers, potential drug targets, or repurposing approved drugs in drug-resistant cancers. Drug repositioning is the approach for using existing drugs for a new therapeutic purpose; it is a highly efficient and low-cost process. To improve current understanding of the MDR-1-related drug resistance in CRC, we explored gene co-expression networks around ABCB1 gene with different network sizes (50, 100, 150, 200 edges) and repurposed candidate drugs targeting the ABCB1 gene and its co-expression network by using drug repositioning approach for the treatment of CRC. The candidate drugs were also assessed by using molecular docking for determining the potential of physical interactions between the drug and MDR1 protein as a drug target. We also evaluated these four networks whether they are diagnostic or prognostic features in CRC besides biological function determined by functional enrichment analysis. Lastly, differentially expressed genes of drug-resistant (i.e., oxaliplatin, methotrexate, SN38) HT29 cell lines were found and used for repurposing drugs with reversal gene expressions. As a result, it is shown that all networks exhibited high diagnostic and prognostic performance besides the identification of various drug candidates for drug-resistant patients with CRC. All these results can shed light on the development of effective diagnosis, prognosis, and treatment strategies for drug resistance in CRC.

## Introduction

Colorectal cancer (CRC) is one of the most lethal types of cancers commonly seen in both men and women. This cancer affects the large intestine in the digestive tract. It is also known as bowel cancer, rectal cancer, and colon cancer. According to the American Cancer Society research, one out of every 23 women in the USA and one out of every 21 men is exposed to this disease (1). According to the database published by the International Agency for Research on Cancer, CRC is the third most common type of cancer found in Turkey and worldwide (GLOBOCAN, 2018) (2). Approximately three-quarters of patients are diagnosed with limited diseases on the intestinal wall or surrounding lymph nodes. Moreover, survival rates and treatment options for CRC may vary depending on a variety of factors including tumor size, location of tumor, and stage of cancer (3).

Over the years, several drugs are developed for the treatment of CRC such as 5-flurouracil, monoclonal antibodies (i.e., bevacizumab, cetuximab) (4), and also combination with 5-fluorouracil/leucovorin with either oxaliplatin (FOLFOX) or irinotecan (FOLFIRI) (5). However, the patients who have advanced CRC are resistant to 5-flurouracil (6). Meanwhile, the development of drug resistance is assumed as one of the major obstacles for cancer therapy. P-glycoprotein (P-gp) (also known as MDR1) is a well-identified membrane transporter protein expressed by the ABCB1 gene. The expression of Multidrug Resistance 1 (MDR1) protein causes chemotherapy failure owing to the efflux of drug molecules out of the cancer cell. MDR1 decreases the drug concentration and causes drug resistance in *in vitro* experiments (7), and high-level MDR1 protein expression was found in several cancer types such as liver, kidney, and colon (8).

Cancer cells upregulate P-gp expression as an adaptive response to evade chemotherapy-mediated cell death. Not all discovered P-gp inhibitors have passed all phases of the clinical trials (9). Therefore, there is a crucial need for efficient treatment strategies to deliver the best possible medical treatment. Network-based approaches can be used to determine specific biomarkers, potential drug targets, or repurposing approved drugs in drug-resistant cancers (10).

Drug repositioning (DR) is the most promising method for all major problems. DR is the approach for using existing drugs for a new therapeutic purpose; it is a highly efficient and low-cost process (11). Existing drugs have already been approved and have been successful in clinical trials, thus potentially reducing the risk of failure in new cases. Recent reviews mentioned repositioning efforts for drug-resistant CRC (12). Drugs such as citalopram, amantadine, and captopril are repurposed for disease prevention or treatment (13–15). Among them, there are also computational efforts to repurpose drugs for CRC, such as the employment of Functional Module Connectivity Map that repurposes GW-8510, etacrynic acid, ginkgolide A, and 6-azathymine (16).

The aim of this research is to repurpose candidate drugs targeting the MDR1 protein and its co-expression network by using a drug repositioning approach for the treatment of CRC. We first constructed co-expression networks around ABCB1 gene within different network sizes consisting of 50, 100, 150, and 200 edges. We performed drug repositioning by using the reversal of co-expression signatures in CRC for all four co-expression networks. The candidate drugs were also assessed by using molecular docking for determining the potential of physical interactions between the drug and MDR1 protein as a drug target. We also evaluated these four networks whether they show diagnostic or prognostic features in CRC. Lastly, the gene expression profiles of drug-resistant (i.e., oxaliplatin, methotrexate, and SN38) HT29 cell lines were used for repurposing drugs with reversal gene expressions. We showed common drug candidates among the candidates found by using co-expression networks around ABCB1 and the candidates that have reversal gene expression profile in the drug-resistant HT29 cell lines.

## Method

### Construction of Co-expression Networks

Four co-expression networks including different degrees (edges 50, 100, 150, and 200) of CRC-specific genes which had co-expression with ABCB1 gene were constructed through the data from TCSBN database (17). The parameters for the minimum and the maximum number of nodes (min-1 max-200) were taken as 50 and 200, respectively; the edge pruning parameter (–log_10_ P) (min-0 max-50) was set to 2. Genes in the networks were classified as downregulated (negative correlation) and upregulated (positive correlation) groups based on the score obtained from the database. In addition, transcription factors (TFs) were determined on the co-expression network. The visualization of networks was constructed using Cytoscape, version 3.7.0 (18).

### Gene Set Over-Representation Analyses

The over-representation analyses were performed through the ConsensusPathDB database (19) to identify functional annotations of molecular pathways significantly associated with four gene groups co-expressed with ABCB1 gene. For this analysis, Kyoto Encyclopedia of Genes and Genomes (KEGG) (20), Reactome (21), and BioCarta (22) were preferred as the primary pathway sources. *P*-values for the pathway analysis were calculated with Fisher's exact test and adjusted *p*-values were obtained using Benjamini–Hochberg correction (23). The results having adjusted *p* < 0.05 were considered statistically significant.

### Identification of Drug Candidates Through Signature-Based Drug Repositioning

Drug repositioning analysis was performed by targeting genes within the co-expression networks via L1000CDS2 (24) search tool including the Library of Integrated Network-based Cellular Signatures (LINCS) L1000 data (25) by using the knowledge about downregulation and upregulation of genes. Negative correlations (score <0) of genes in networks were used as downregulated genes, whereas positive correlations (score >0) of genes were used as upregulated genes (Supplementary Table 1). The genes within co-expression with *p* < 10^−5^ were considered as significant to investigate the drugs. Drug candidates indicating potentially reverse effects for genes in the co-expression network were determined to the treatment of CRC.

### Molecular Docking Protocol

The structure of ABCB1 protein was obtained from Protein Data Bank (PDB) (26) with a PDB code of 6QEX and the structures of candidate drugs were obtained from the PubChem database (27). Binding residues including 11 phenylalanine (F72, F303, F314, F336, F732, F759, F770, F938, F942, F983, F994), two leucine (L339, L975), one isoleucine (I306), and one methionine (M949) in the transmembrane domain (TMD) of ABCB1 were taken into account for molecular docking analyses (28), which were carried out through AutoDock Vina software (29) to bind the structures of candidate drugs to binding residues of ABCB1 (TMD). In addition, ligands were also docked to two nuclear-binding domains (NBD) of ABCB1 (30). Binding affinities were detected to determine the significance of the binding after molecular docking.

### Collection and Evaluation of Drug-Resistant Data Sets

Data sets related to the drug resistance in CRC were scanned within literature, and two microarray data sets were found to include control cells and methotrexate-, oxaliplatin-, and SN38-resistant cells (31) because ABCB1 is associated with the resistance to these drugs (32–34). Differentially expressed genes (DEGs) were statistically detected in all drug-resistant microarray data sets through the previously published pipeline (35, 36). This pipeline was followed by data normalization with Robust MultiArray Average (37), hypothesis testing by linear models for microarray data (LIMMA) method (38), and controlling the false discovery rate through the Benjamini–Hochberg method (23). DEGs were selected as significant based on adjusted *p* < 0.05 and downregulated and upregulated genes were determined by the aid of fold change (FC) values with threshold |log2FC| ≥1. Candidate drugs were identified with drug repositioning through the L1000-CDS2 tool based on downregulation or upregulation information. Candidate drugs for each drug-resistant cell line are listed in Supplementary Table 2.

### Diagnostic and Prognostic Features of ABCB1 Mediated Co-expression Networks

Principal component analyses (PCA) were performed to each of four gene groups co-expressed with ABCB1 gene. For this purpose, gene expression profiles of FPKM normalized RNA-Seq data set with 644 primary colorectal tumor and 51 matched normal samples were obtained from TCGA. Each simulation was carried out with the randomly chosen 50 normal and 50 tumor samples which have expression data of genes co-expressed with ABCB1 gene. The first two principal components explaining at least 80% of total variance were considered to identify sensitivity and specificity metrics.

Survival analyses were carried out according to the well-established pipeline (39, 40) using FPKM normalized RNA-Seq data originated TCGA data set for determination of the prognostic performance of each edge. The subjects were partitioned into low-risk and high-risk groups according to their prognostic index (PI), also known as the risk score, which is the linear component of the Cox model (PI = β1x1 + β2x2 +… + βpxp, where xi is the expression value of each gene and βi is the coefficient obtained from the Cox fitting). All analyses were performed using the “survival” package in R (version 3.6.1) (https://www.R-project.org/). Kaplan–Meier plots were used for the evaluation of the survival signatures in each edge, and the cut-off for the log-rank *p*-value was considered as <0.05 to describe statistical significance. Hazard ratio (HR = O_1_/E_1_/O_2_/E_2_) was calculated for the determination of the significance of the survival plots according to the rate between the relative death rate in group 1 (O_1_/E_1_) and the relative death rate in group 2 (O_2_/E_2_), where O represents the observed number of deaths and E represents the expected number of deaths.

## Results

### ABCB1 Mediated Co-expression Networks as Resistance Signatures of CRC and Their Biological Functions

Co-expression networks are one of the major network approaches indicating altered co-expression patterns of genes between two phenotypes. These networks represent significant potential to identify gene clusters associated with the phenotype of interest in many cancer types (41, 42).

**Figure 1:**
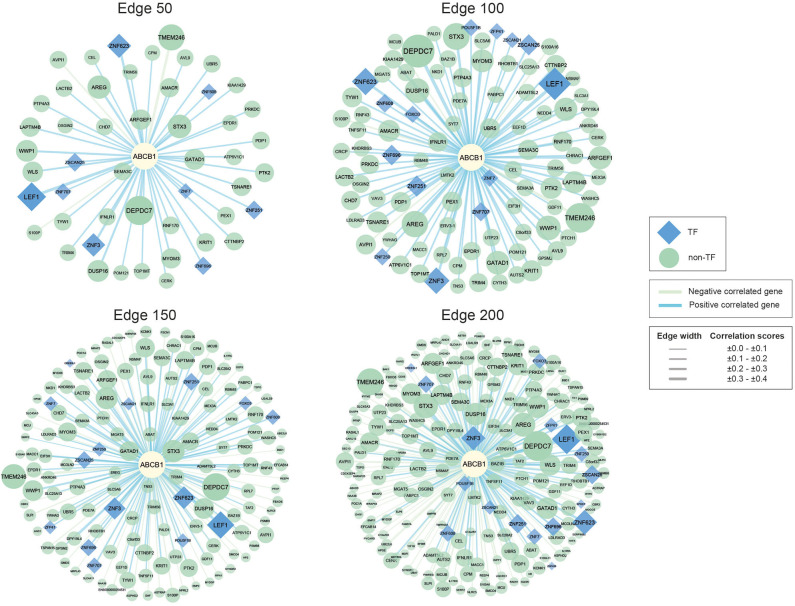
Networks of ABCB1 co-expressed genes. Negatively correlated genes have green edges, whereas positively correlated genes have blue edges. Transcription factors are represented as blue diamonds and edge thickness varies according to the score of the correlation between genes, whereas node size varies based on the *p*-value of the correlated genes. Transcription factors are represented as blue diamond, and edge thickness varies according to the score of the correlation between genes whereas node size varies according to the statistical significance (i.e., *p*-value of the correlated genes).

In our study, we used co-expression networks around ABCB1 gene to elucidate potential drugs for avoiding drug resistance in CRC. For that purpose, we reconstructed four co-expression networks including different degrees (edges 50, 100, 150, and 200) of CRC-specific genes that demonstrated significant co-expression patterns with ABCB1 gene through the data from TCSBN database (Figure 1).

The reconstructed networks were named according to network size (i.e., edges 50, 100, 150, and 200). As the number of interactions increased, the number of negatively correlated genes increased in parallel. In edge 50, 3 negative correlations, 47 positive correlations; in edge 100, 5 negative correlations, 95 positive correlations; in edge 150, 49 negative correlations, 101 positive correlations; and in edge 200, 100 negative correlations, 100 positive correlations were observed.

To elucidate the regulatory mechanisms behind the co-expression patterns of module genes and to evaluate the condition-specific expression pattern alterations, we performed serial analyses to link the key regulators of the networks. Transcription factors were already existing with the networks; 9 of the genes were encoding TFs in edge 50, 14 of the genes were encoding TFs in edge 100, 15 of the genes were encoding TFs in edge 150, and 17 of the genes were encoding TFs in edge 200 (Supplementary Table 3).

**Figure 2:**
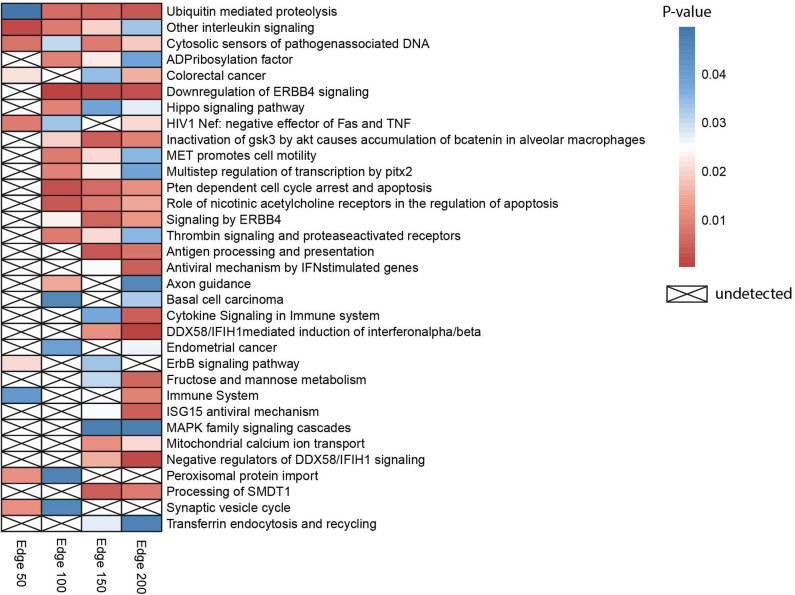
Heatmap indicating biological pathways in which co-expressed genes involved based on the over-representation analysis. Each cell on the heatmap was colored according to the *p*-value of genes in each co-expression network. If the pathway is not detected, it was indicated with a cross.

Gene set over-representation analysis of the edge 50, 100, 150, and 200 networks were performed to determine significant biological pathways comprising these gene sets (Figure 2). The common pathways among all the number of co-expressed genes were ubiquitin-mediated proteolysis, interleukin signaling, and cytosolic sensors of pathogen-associated DNA. Particularly, interleukin signaling has been reported as involving multidrug resistance of different cancer types as well as CRC (43).

Besides, the pathway related to CRC was detected when the three different numbers of edges were examined. The pathway ubiquitin-mediated proteolysis, antigen processing and presentation, MAPK family signaling cascades, ErbB signaling pathway, and Hippo signaling pathway were also previously reported as pathways associated to drug resistance (44).

**Figure 3:**
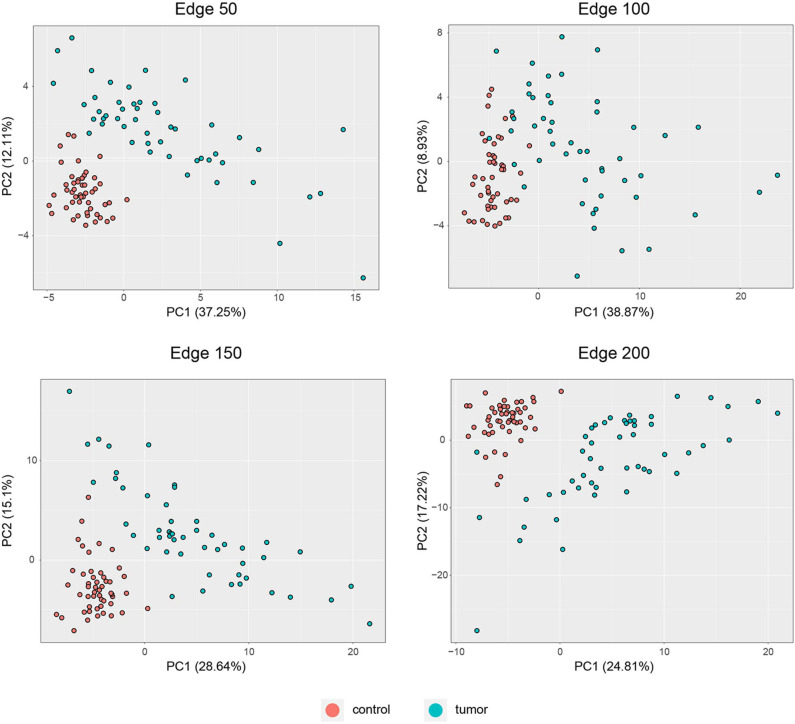
PCA plots for each co-expression network. Red dots represent healthy colorectal samples, whereas blue dots represent colorectal tumor samples. Variances explained by the principal components are indicated in axis titles for all plots.

PCA was performed to identify the diagnostic performance of the correlated genes. As shown in Figure 3, the correlated genes demonstrated promising performance to be considered for diagnostic purposes. These genes exhibited distinctive features between 50 control and 50 tumor samples. Sensitivity, specificity, and diagnostic odds ratio (DOR) metrics were calculated considering the two most significant principal components. Sensitivity and specificity values were determined as 100 and 100% for edge 50, 9% and 96% for edge 100, 100 and 100% for edge 150, and finally 98 and 100% for edge 200. All gene sets exhibited high diagnostic performance (sensitivity ≥0.92, specificity ≥0.98, DOR ≥276).

**Figure 4:**
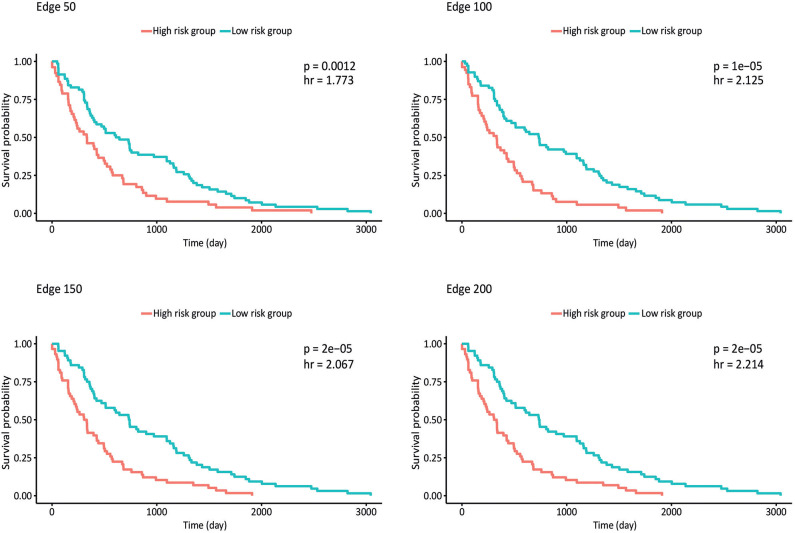
Kaplan–Meier plots estimating survival of patients for correlated networks indicating *p*-value and hazard ratio on each curve.

Survival analysis was also performed by partitioning patients into low-risk and high-risk groups according to the expression levels of each gene in the network, and *p*-values and hazard ratios (HRs) were found to evaluate the prognostic power for all networks (Figure 4). In edge 50, *p*-value and HR were 0.0012 and 1.773, respectively. As the network size increased, both the statistical significance and the HRs were improved. Edge 200 presented a *p-*value and HR of 2 × 10^−5^ and 2.214, respectively.

### Identification of Potential Repositioning Drugs and Molecular Docking Simulations

By reconstructing the ABCB1 gene–mediated co-expression networks, our main goal was to identify the potential therapeutic targets and candidate drugs. Through employing a drug repositioning methodology, here, we aimed to propose hypotheses with drugs used outside of oncology but tentatively promising in cancer as in previous studies (45, 46).

**Figure 5:**
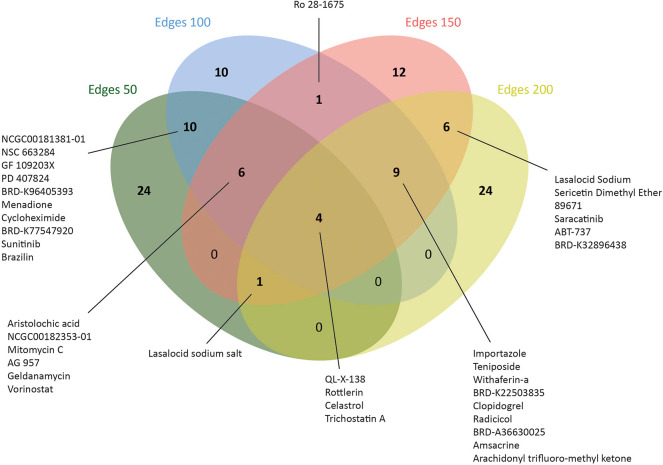
Number of drugs that were identified via drug repositioning for each co-expressed network.

Therefore, L1000CDS2 was used to find appropriate drugs with reversal effects on the gene expression profiles in CRC. As a result, 45 drugs for edge 50, 41 drugs for edge 100, 39 drugs for edge 150, and 44 drugs for edge 200 were determined (Figure 5 and Supplementary Table 4). Eight of the candidate drugs were antineoplastic agents, such as celastrol, rottlerin, withaferin A, amsacrine, teniposide, geldanamycin, sunitinib, and vorinostat, which are known for the treatment of various diseases such as acute lymphoblastic leukemia, stomach cancer, and pancreatic cancer; two of the potential drugs (arachidonyl trifluoro-methyl ketone and cycloheximide) were neuroprotective agents. Furthermore, seven drugs (importazole, AG 957, NCGC00182353-01, brazilin, NCGC00181381-01, PD 407824, and Ro 28-1675) with inhibitor activity were also proposed as novel candidates for CRC in this study. Among those, importazole is a transport receptor importin-β (47) and AG 957 is a protein tyrosine kinase inhibitor (48). Moreover, brazilin is a NF-kappaB inhibitor (49) and PD 407824, a hepatoprotective agent, is known as Wee1/Chk1 inhibitor (50), whereas Ro 28-1675 is known as glucokinase activator (51). However, the action mechanisms of NCGC00182353-01 and NCGC00181381-01 were not identified yet (Supplementary Table 5).

The traditional path of drug development, that is, finding a proper drug target, identifying a potential drug, and setting up experimental assays to test the efficiency of the proposed drug, is a time-consuming and high-cost process. However, molecular docking, which simulates the binding affinity of a drug in three-dimensional structure of a drug target, can be implemented as a higher resolution simulation method (52) and is accepted as the most useful method to predict the binding affinity of the drug–target complex with minimum free energy (53).

**Figure 6:**
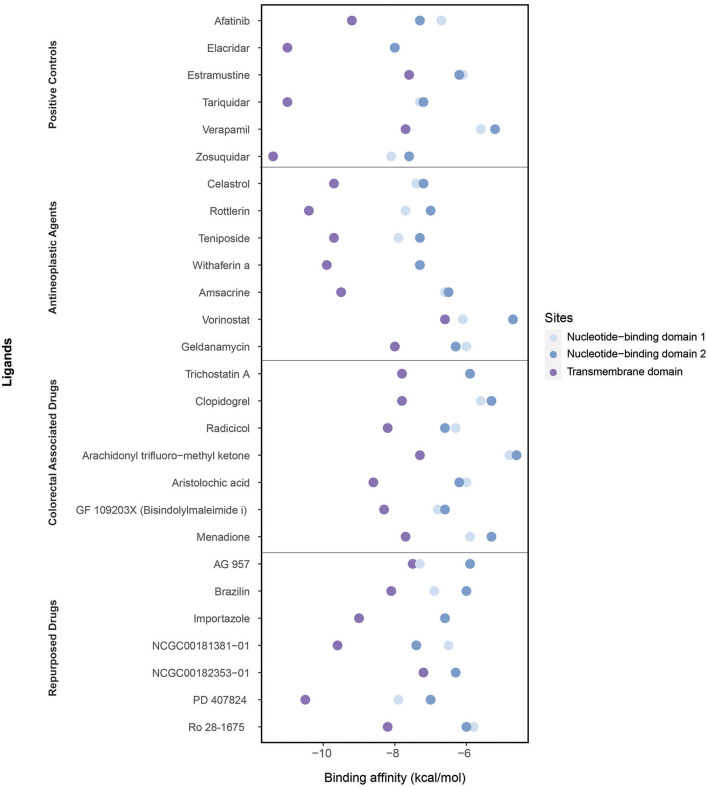
Binding affinities of ligands (the positive controls, antineoplastic agents, colorectal associated, and repurposed drugs) estimated through the molecular docking simulations considering three domains: the nucleotide-binding domain 1, nucleotide-binding domain 2, and transmembrane domain.

We performed molecular docking simulations on three different domains of ABCB1: the ATP binding sites (NBD1 and NBD2) and TMD, which is the site of substrate recognition and translocation (54). The binding activities estimated by molecular docking simulations are shown in Figure 6.

As the positive controls, the well-known ABCB1 inhibitors afatinib, elacridar, estramustine, tariquidar, verapamil, and zosuquidar were used. Afatinib, estramustine, and zosuquidar were antineoplastic agents, elacridar was an acridonecarboxamide derivative, tariquidar was an anthranilamide derivative, and verapamil was an antihypertensive calcium blocker channel. The binding affinities of afatinib, elacridar, tariquidar, and zosuquidar on TMD were −9.2, −11, −11, and −11.4 kcal/mol, respectively, which were significantly high compared with those for estramustine (−7.6 kcal/mol) and verapamil (−7.7 kcal/mol). Although the binding affinities of the ligands were lower in NBDs (when compared with those in TMD), the highest results were obtained in elacridar (NBD1: −8 kcal/mol, NBD2: −8 kcal/mol), tariquidar (NBD1: −7.3 kcal/mol, NDB2 −7.2 kcal/mol), and zosuquidar (NBD1: −8.1 kcal/mol, NBD2: −7.6 kcal/mol) (Supplementary Table 6). On the contrary, while focusing on the antineoplastic agents, we obtained the high binding affinities by docking at TMD with the colorectal associated drugs and repurposed drugs (Supplementary Table 4). Overall, TMD came into prominence as a promising site for ligand binding to inhibit ABCB1.

**Figure 7:**
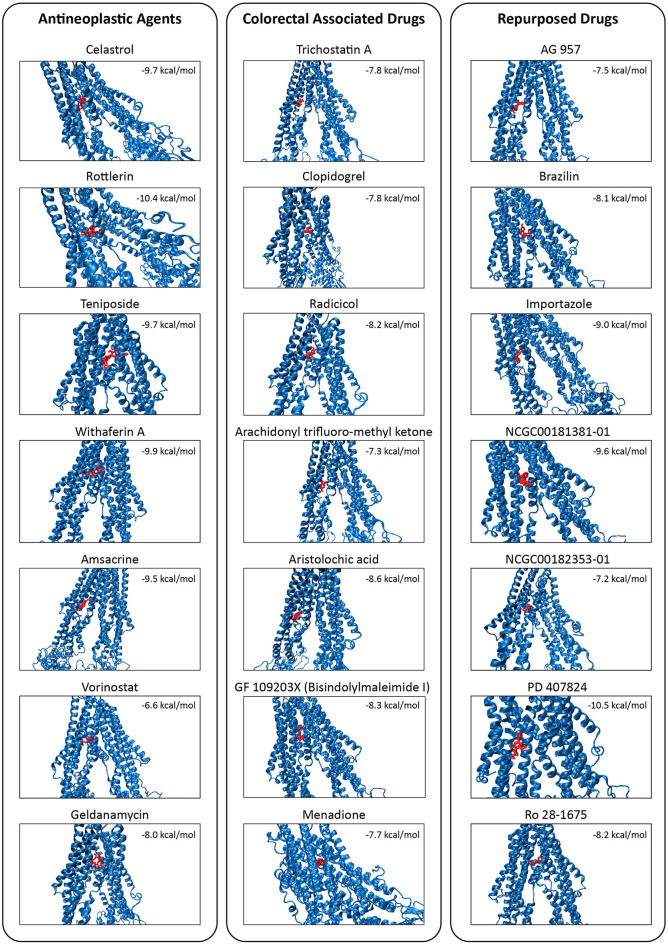
Molecular docking simulations of the candidate drugs (shown in red) on TMD of the MDR1 protein (shown in blue). Binding affinity for each protein-drug docking was indicated.

The high binding affinities of these drugs were rottlerin (−10.4 kcal/mol), withaferin A (−9.9 kcal/mol), teniposide (−9.7 kcal/mol), and celastrol (−9.7 kcal/mol) in antineoplastic agents section; aristolochic acid (−8.6 kcal/mol), GF 109203X (−8.3 kcal/mol), and radicicol (−8.2 kcal/mol) inside of colorectal associated section; and finally when we look at the novel drugs, the high binding affinities of these drugs were PD 407824 (−10.5 kcal/mol), NCGC00181381-01 (−9.6 kcal/mol), importazole (−9 kcal/mol), Ro 28-1675 (−8.2 kcal/mol), and brazilin (−8.1 kcal/mol) in repurposed drugs section. As a comparison of these novel drugs with positive controls, brazilin, importazole, NCGC00181381-01, PD 407824, and Ro 28-1675 showed that the importance of repurposed drugs reversing the effect of caused by ABCB1 (Figure 7). Moreover, it was investigated that the targetability of co-expressed TFs that regulate ABCB1 by the potential drugs with possible drug-resistant reversal effect in ABCB1. the protein structures were investigated for 17 TFs determined on the co-expression network, however, only two of them (FOXO3 and SPDEF) were found as having a structure for molecular docking analysis. All drugs indicated in Figure 7 were docked on these two TFs and binding affinities were given in Supplementary Table 7.

### The Cross-Validation of Repurposed Drugs Using Transcriptomic Codes of Drug-Resistant HT29 Cells

**Figure 8:**
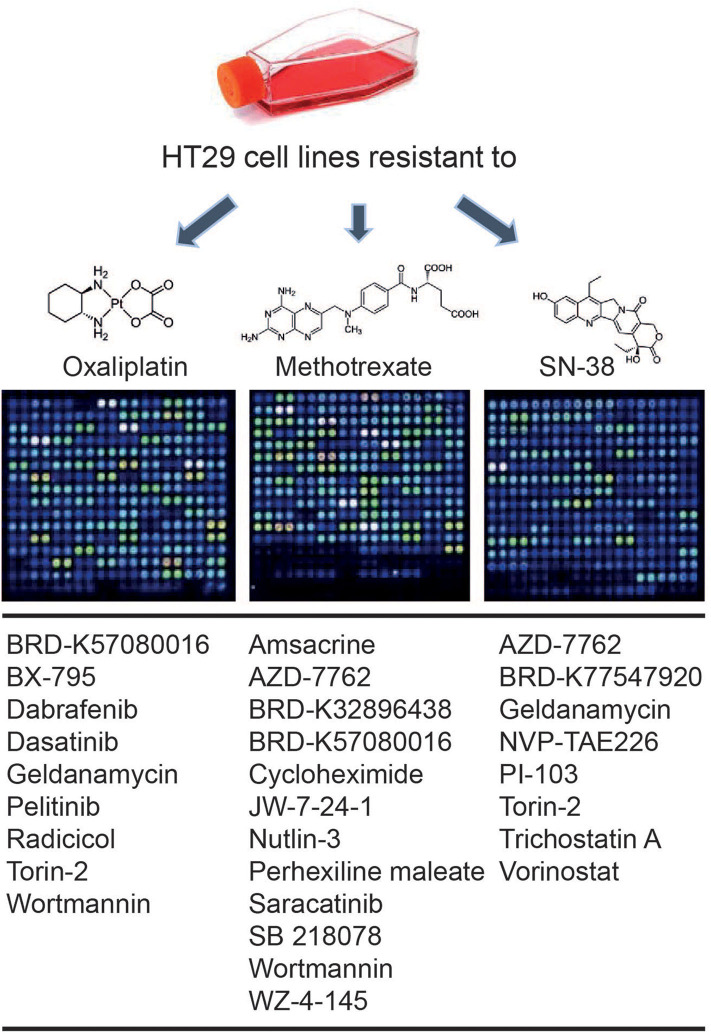
Drugs identified throughout the study in both methods: drug repurposing through (1) ABCB1 mediated co-expression networks and (2) gene expression profiling in drug-resistant colorectal cancer cell lines.

An *in silico* cross-validation study was performed using transcriptome data for genes co-expressed with ABCB1 in drug-resistant HT29 cells. We acquired transcriptome datasets of oxaliplatin-resistant, methotrexate-resistant, and SN38-resistant HT29 cancer cell lines (Table 1). After analyzing DEGs in resistant cell lines, we queried these gene expressions for drug repositioning by L1000-CDS2 as same as we performed within co-expressed modules. All drugs offering reversal expression patterns against drug-resistant cell lines are listed in Supplementary Table 2. A comparison of these drugs with the drug candidates previously identified through co-expression networks presented common signatures as illustrated in Figure 8.

**Table 1:** Information about the data sets used for the identification of DEGs and candidate repurposed drugs.

**Data set**	**Drug resistance**	**Control Tumor sample**	**Drug-resistant Tumor sample**
GSE16648	Methotrexate	3 sensitive cells	3 methotrexate-resistant cells
GSE42387	Oxaliplatin	3 control parental cells	3 oxaliplatin-resistant cells
GSE42387	SN38	3 control parental cells	3 SN38-resistant cells

## Discussion

Drug resistance in cancer becomes more challenging in cancer therapy today. There are many underlying mechanisms of resistance parallel to the number of patients with cancer because each cancer case has its characteristics leading to various tumor progression or eventually death (55).

As same as others, drug-resistant colon cancer is still an obstacle for effective therapy and novel therapeutic strategies are urgently needed. Hence, investigation on the underlying mechanisms conferring drug resistance, as well as the development of safe and effective reversing agents by targeting these mechanisms, will play a pivotal role in the successful chemotherapy for colon cancer (56). To improve the current understanding of the MDR-1-related drug resistance in colorectal cancer, we explored gene co-expression networks around ABCB1 gene and repurposed candidate drugs for the treatment of CRC. The candidate drugs were also assessed by using molecular docking for determining the potential of physical interactions between the drug and MDR1 protein as a drug target. We also evaluated the diagnostic or prognostic features of co-expressed gene networks in CRC. Considering gene expression profiles in drug-resistant (i.e., oxaliplatin, methotrexate, SN38) HT29 cell lines, another drug repositioning strategy was applied to identify drugs that demonstrate a reversal effect on the transcriptomic reprogramming in CRC. All these results can shed light on the development of effective diagnosis, prognosis, and treatment strategies for drug resistance in CRC.

As one of the reasons for the resistance problem in patients with cancer, MDR-1 protein has been chosen as a target in this study. To improve the current understanding of the MDR-1-related drug resistance in CRC, we explored gene co-expression networks around ABCB1 gene encoding the MDR-1 protein. Disease co-expression networks in cancer are mainly used to explore systems biomarkers for exploring prognosis and treatment strategies (41, 42, 57).

Moreover, co-expression networks in different cancer types were applied not only for insights in cancer biology but also for drug repositioning (58). In this study, we outline a roadmap for tackling the problem of resistance by using co-expression networks for the first time to the best of our knowledge.

Our approach differs from previous drug repositioning studies in several respects. First, we use ABCB1 mediated co-expression network with different edge numbers which enable robust and sensitive detection of gene co-expression modules even around this protein whereas previous studies were only focused on MDR-1 protein as a drug target to overcome drug-resistance problem (59, 60).

Second, we applied the drug repositioning strategy by seeking a reversed expression effect of the disease condition. In previous studies, the differentially expressed genes were determined and used as a query for determining reversal expression patterns induced by drugs (45, 46, 61). Our input data for drug repositioning has an essential difference although we utilized a familiar strategy. Instead of using differential expressed genes, we employed positively and negatively correlated genes for ABCB1 protein as a proxy.

Lastly, our approach is independent of healthy samples and only focused on CRC-associated transcriptome data. Several previous studies focused on co-expression patterns in cancer as opposed to the normal transcriptome represented by healthy tissue samples. Although intuitive, such a strategy is prone to exclusion of disease-related modules that only superficially resemble normal ones (62, 63).

It should be noted that co-expression studies focusing on only a subset of genes rather than the entire transcriptome has an advantage from a computational perspective into translational science. This is caused by the fact that large networks are technically challenging for translational science into the clinic. Therefore, the use of robust co-expression modules in drug repositioning or biomarker studies is also another promising way of the current study.

Our findings emphasize the value of studying co-expression modules to overcome drug resistance and to exploit this knowledge for suggesting potential therapeutics. Such an approach is fundamentally different from the currently common drug repositioning studies with its starting point. Results can be employed either determining mechanisms underlying MDR-1 mediated drug resistance in CRC or it can be exploited to anticipate the new repurposed drugs for the treatment. Moreover, it can provide a complementary strategy for biomarker discovery in drug-resistant cancers as well as therapy options.

## Data Availability Statement

The datasets presented in this study can be found in online repositories. The names of the repository/repositories and accession number(s) can be found in the article/Supplementary Material.

## Author Contributions

BT designed the study. HB and GG performed the analyses and wrote the manuscript. BT, AM, and KA revised and contributed to the manuscript. All authors contributed to the article and approved the submitted version.

## Conflict of Interest

The authors declare that the research was conducted in the absence of any commercial or financial relationships that could be construed as a potential conflict of interest.
